# Associations between Autonomic Function and Cognitive Performance among Patients with Cerebral Small Vessel Disease

**DOI:** 10.3390/brainsci13020344

**Published:** 2023-02-17

**Authors:** Guoliang Hu, Jean-Paul Collet, Mengxi Zhao, Yao Lu, Yilong Wang

**Affiliations:** 1 Department of Neurology, Beijing Tiantan Hospital, Capital Medical University, Beijing 100070, China; 2 China National Clinical Research Center for Neurological Diseases, Beijing 100070, China; 3 Advanced Innovation Center for Human Brain Protection, Capital Medical University, Beijing 100070, China; 4 National Center for Neurological Diseases, Beijing 100070, China; 5 Department of Medicine, The University of British Columbia, Vancouver, BC V6T 1Z4, Canada; 6 Chinese Institute for Brain Research, Beijing 102206, China

**Keywords:** autonomic function, heart rate variability, cognitive performance, MMSE, MoCA, cerebral small vessel disease, patients

## Abstract

Data linking heart rate variability (HRV) and cognitive status remains controversial and scarce, particularly in cerebral small vessel disease (CSVD) patients. Whether the association between HRV and cognitive performance exists in CSVD patients is unclear. Hence, we aimed to investigate the association between HRV and cognitive performance in patients with CSVD. This cross-sectional study was conducted among 117 CSVD patients. All patients underwent HRV assessment and global cognitive evaluation by the Mini-Mental-State Examination (MMSE) and Montreal Cognitive Assessment (MoCA). Multivariable analyses were performed to evaluate the association between HRV and cognitive status. The mean age of study population was 59.5 ± 11.8 years and 39.3% were female. After adjusting for confounding factors, a higher high frequency (HF) norm was independently associated with better MMSE scores (β = 0.051; 95% confidence interval (CI): 0.012~0.090; *p* = 0.011) and MoCA scores (β = 0.061; 95% CI: 0.017~0.105; *p* = 0.007), while a higher low frequency (LF)/HF ratio was independently associated with worse MMSE scores (β = −0.492; 95% CI: −0.893~−0.092; *p* = 0.017) and MoCA scores (β = −0.691; 95% CI: −1.134~−0.248; *p* = 0.003). The HF norm was positively associated with global cognitive performance, whereas the LF/HF ratio was negatively associated with global cognitive performance among CSVD patients. Further study of the relationship between autonomic function and cognitive performance is warranted.

## 1. Introduction

Globally, more than 40 million people currently live with dementia, and this number is expected to almost double every 20 years [1,2]. As a global health burden, cognitive impairment poses a significant challenge to affected individuals, families, and healthcare systems [1,3,4]. Cerebral small vessel disease (CSVD), a common neuroimaging condition among elderly individuals characterized using the Standards for Reporting Vascular Changes on Neuroimaging (STRIVE) [5], is now recognized as a significant vascular contributor to cognitive decline [6,7]. Given the current aging population with a high prevalence of CSVD [8,9], it is of great significance to identify the novel early biomarkers of cognitive impairment to improve targeted strategies for preventing or intervening in dementia during the preclinical phase.

Heart rate variability (HRV) is a beat-to-beat alteration in the normal sinus rhythm resulting from the constant interaction between the sympathetic and parasympathetic arms of the autonomic nervous system [10,11]. HRV measured using electrocardiogram (ECG) recordings is used as a standard parameter of autonomic function and is correlated with the risk factors of cognitive impairment (e.g., hypertension, diabetes mellitus, and subclinical inflammation) [12,13,14], which indicates that HRV and cognitive impairment have common risk factors. Previous findings regarding the relationship between autonomic function and cognitive performance remain controversial [15,16,17,18] in spite of the well explored association between autonomic dysfunction and increased cardiovascular morbidity and mortality [19], abnormal prefrontal cortical activity [20], and reduced regional cerebral blood flow [21]. A multi-ethnic cohort study conducted among 3018 middle-aged and elderly adults from the Multi-Ethnic Study of Atherosclerosis showed that higher HRV measures were prospectively (β = 0.37, 95% CI: 0.06, 0.67) and cross-sectionally (β = 0.31, 95% CI: 0.04, 0.59) associated with better cognitive performance, independent of cardiovascular risk factors and diseases [16]. Moreover, the Women’s Health and Aging Study I showed that autonomic dysfunction, particularly decreased parasympathetic activity, is independently associated with global cognitive impairment (odds ratio = 6.74, 95% CI: 2.27, 20.0) in community-dwelling women [17]. However, recent data from the elderly population showed that increased sympathetic activity is significantly associated with better cognitive performances (MMSE: β = 0.416, *p* = 0.028; MoCA: β = 0.613, *p* = 0.001) [18]. The contrary results indicate that the balance of autonomic system rather than the function of each sub-system may play a more important role in regulating cognitive function. Further observational studies are needed to clarify this issue.

Moreover, although numerous studies have evaluated the relationship between autonomic function and cognitive performance, data linking HRV and cognitive performance in patients with CSVD remain scarce. Patients with CSVD are at high risk for cognitive impairment and vascular dementia, possibly due to disruption of structural and functional network connections [6]. Whether the association between HRV and cognitive status exists in patients with CSVD remains unclear. Therefore, we aimed to investigate the association between HRV and global cognitive performance in patients with CSVD.

## 2. Materials and Methods

### 2.1. Subject Recruitment

Patients with CSVD were consecutively recruited from the Neurology Department of Beijing Tiantan hospital between 6 January 2020 and 28 March 2022. This study was approved by the Ethics Committee at Beijing Tiantan Hospital (approval number: KY201914002, approval date: 29 December 2019). All patients had typical magnetic resonance imaging (MRI) features of CSVD. To qualify for enrollment, patients had to meet the following inclusion criteria: (1) patients aged 18 years or older [22]; (2) patients who underwent MRI and had at least one of the typical MRI features of CSVD [23,24]: white matter hyperintensity (WMH) Fazekas scores ≥2 [22], WMH Fazekas scores = 1 combined with at least one lacune or at least two cardiovascular risk factors (including hypertension, dyslipidemia, diabetes mellitus, obesity, current smoking, or history of vascular events except for stroke), or recent subcortical lacunar infarction [22]; (3) patients with independent ability in activities of daily life (modified Rankin Scale<= 2) [22]; (4) informed consent from patients or legally authorized representatives. Patients who had diffusion-weighted imaging signs of recent cerebral infarction (diameter >20 mm), acute hemorrhagic stroke, acute subarachnoid hemorrhage, previous history of cerebrovascular malformation, aneurysmal subarachnoid hemorrhage or untreated aneurysms (>3 mm in diameter), dementia caused by diagnosed neurodegenerative disease (e.g., Alzheimer’s disease, Parkinson’s disease), presence of non-vascular white matter lesions, and diagnosed mental disorders according to the DSM-V were excluded from the study [22]. At least two neurologists reviewed the clinical manifestations, MRI features, and other diagnostic test results before making the diagnosis of CSVD.

### 2.2. HRV Assessments

All eligible patients received 6-lead, 15-min ECG recording after their enrollment. HRV was computed using time-domain and frequency-domain analysis of 3 consecutive, 5-min resting ECGs obtained by trained technicians using Acqknowledge 4.2.1 software (BIOPAC Systems, Inc., Goleta, CA, USA). ECGs were obtained in the seated position. Artifacts and arrhythmia were detected and removed using visual inspection methods and correction filters provided and incorporated from the Acqknowledge 4.2.1 software. HRV was quantified using time-domain and frequency-domain parameters.

In the time domain, the following parameters were calculated: the standard deviation of the normal/normal intervals (SDNN, in millisecond [ms]) and the root mean squares of successive differences of normal/normal intervals (RMSSD, in ms). In particularly, SDNN represents total variability and thus joint sympathetic and parasympathetic modulation of HRV, whereas RMSSD reflects parasympathetic activity [11].

In the frequency domain, the power spectra of the following frequency bands were calculated: high frequency norm (HF norm, normalized unit, in n.u.), and the low frequency/high frequency ratio (LF/HF ratio). The HF norm and the LF/HF ratio are considered as an index of parasympathetic modulation and an index of global sympatho-vagal balance, respectively [18].

### 2.3. Clinical and Cognitive Function Assessment

Demographic and clinical information were extracted from original medical records and patient self-report, including vascular risk factors, medical history, and medication use (for definitions, see the Supplementary Methods).

Global cognitive function was measured by trained researchers in a quiet environment using the Mini-Mental-State Examination (MMSE) and the Montreal Cognitive Assessment (MoCA). The total score ranges from 0 to 30, with higher scores indicating better cognitive functioning. Based on the MMSE score and education level, cognitive impairment was defined as an MMSE score of <18 for those without formal education, <21 for those with 1–6 educational years, or <25 for those with over 6 educational years [25,26]. As for the MoCA test, a score of less than 26 is considered as a cognitive impairment [27]. Patients received one additional point if they had 12 years or less of formal school education and a MoCA score of <30 points.

### 2.4. Brain MRI Acquisition and Assessment

All patients underwent brain MRI scans using a 3.0-Tesla MRI scanner (Siemens MAGNETOM Prisma, Erlangen, Germany) with a Siemens 64-channel Prisma head coil based on a standardized protocol. The primary sequences included the following: whole brain T1-weighted images, T2-weighted fluid-attenuated inversion recovery images, diffusion-weighted imaging sequences, and susceptibility-weighted imaging sequences (for parameters, see the Supplementary Methods). Based on the MRI results, two well-trained raters performed an imaging assessment of the typical CSVD markers, blinded to the patient’s clinical information, and according to the STRIVE criteria [5].

### 2.5. Statistical Analyses

Shapiro–Wilk test and QQ plot were conducted to assess the normality of our data (Supplementary Figure S1). Continuous variables with normal distribution are shown as mean (standard deviation [SD]), and differences between groups were compared using *t*-tests; continuous variables with skewed distribution are shown as median (interquartile range [IQR]) and compared using the Wilcoxon rank-sum test; categorical variables are presented as the number (percentage) and compared using chi-square test. Correlation analysis was performed using Spearman’s correlation coefficient. Multivariable linear regression analysis was performed to examine the association between HRV and global cognitive performance, as evaluated using the MMSE and MoCA test. Absolute values of HRV were transformed by natural logarithm before entering the model. Univariate analysis was first performed, and then a multivariable linear regression model was established to adjust for potential confounders, including age, sex, body mass index (BMI), education, hypertension, diabetes mellitus, dyslipidemia, and history of stroke.

Statistical analyses were performed using SAS 9.4 (SAS Institute, Cary, NC, USA) and R software 4.0.1. Two-tailed *p* values of <0.05 were considered statistically significant.

## 3. Results

### 3.1. Baseline Characteristics of Patients

The baseline characteristics of the study population are summarized in Table 1. The mean age of the 117 patients was 59.53 ± 11.79 years; 39.32% were women, and 25.64% completed college. The most frequently reported comorbidities were dyslipidemia (89.74%) and hypertension (81.20%). Almost one-third of the patients (31.62%) were current smokers. Regarding global cognitive assessment, the median of MMSE and MoCA scores were 26.00 and 21.00, respectively. In particular, 41 (35.04%) patients showed impaired cognitive function as defined by the MMSE test, while 98 (83.76%) patients showed impaired cognitive function as defined by the MoCA test.

### 3.2. Univariate Comparisons of HRV According to Different Cognitive Function

Univariate comparisons of HRV parameters according to different cognitive function were shown in Table 2. By comparing the HRV parameters of patients with the normal and impaired cognitive function defined by MMSE scores, we found significant differences in the HF norm (57.23 [42.68, 67.46] vs. 42.95 [29.05, 52.59], *p* < 0.001) and the LF/HF ratio (0.75 [0.48, 1.34] vs. 1.33 [0.90, 2.44], *p* < 0.001), indicating decreased parasympathetic functioning and increased sympathetic functioning in patients with impaired cognitive function. Similar results were observed under the definition of the MoCA scores.

### 3.3. Spearman Correlation between HRV and Global Cognitive Performance

**Figure 1:**
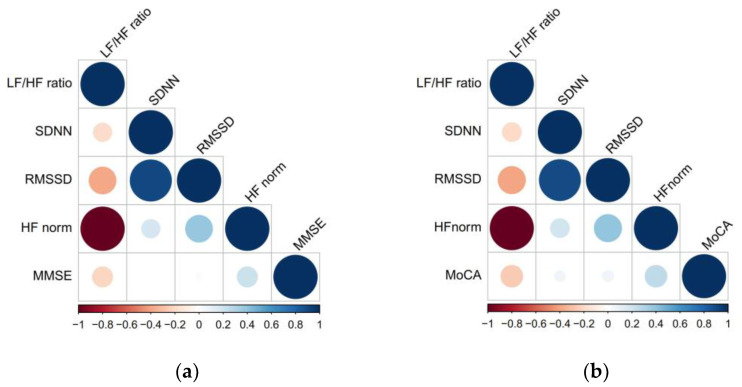
Spearman correlation between HRV parameters and MMSE and MoCA scores among patients with CSVD. (**a**). Spearman correlation between HRV parameters and MMSE scores. (**b**) Spearman correlation between HRV parameters and MoCA scores. CSVD, cerebral small vessel disease; HF, high frequency; HRV, heart rate variability; LF, low frequency; MMSE, Mini-Mental-State Examination; MoCA, Montreal Cognitive Assessment; RMSSD, root mean squares of successive differences of normal/normal intervals; SDNN, standard deviation of the normal/normal intervals.

Spearman correlations between HRV parameters and global cognitive assessment are shown in Figure 1 and Supplementary Table S1. HF norm and LF/HF ratio showed a mild correlation with MMSE (all rs = |0.217|, *p* = 0.019) and MoCA scores (all rs = |0.252|, *p* = 0.006) in all study populations. Similar results were also found in subgroups of the study population, including patients with age <75, patients with hypertension, patients without diabetes and patients without history of stroke (Supplementary Table S2).

### 3.4. Association between HRV Parameters and Global Cognitive Function

The association between HRV and global cognitive function was also tested using multivariable linear regression models and presented as the regression coefficient (β) of the ln-transformed HRV values (Table 3). Univariable analysis showed that the HF norm was associated with better MMSE scores (β = 0.057; 95% CI: 0.010, 0.104; *p* = 0.018), whereas the LF/HF ratio was associated with worse MMSE scores (β = −0.576; 95% CI: −1.058, −0.093; *p* = 0.020). After adjusting for age, sex, BMI, education, hypertension, diabetes mellitus, dyslipidemia, and history of stroke, multiple linear regression analyses showed that a higher HF norm was independently associated with better MMSE scores (β = 0.051; 95% CI: 0.012, 0.090; *p* = 0.011). Conversely, a higher LF/HF ratio was independently associated with worse MMSE scores (β = −0.492; 95% CI: −0.893, −0.092; *p* = 0.017) (Table 3). Subgroup analyses showed that the significant associations between frequency domain parameters and MMSE scores were only observed in patients with age <75 (HF norm: β = 0.059; 95% CI: 0.015, 0.103; *p* = 0.009; LF/HF ratio: β = −0.466; 95% CI: −0.901, −0.030; *p* = 0.036), patients without hypertension (LF/HF ratio: β = −1.195; 95% CI: −2.197, −0.193; *p* = 0.023), patients without diabetes (HF norm: β = 0.066; 95% CI: 0.025, 0.108; *p* = 0.002; LF/HF ratio: β = −0.516; 95% CI: −0.951, −0.081; *p* = 0.021), and patients without history of stroke (HF norm: β = 0.057; 95% CI: 0.017, 0.097; *p* = 0.006; LF/HF ratio: β = −0.420; 95% CI: −0.811, −0.029; *p* = 0.036) (Supplementary Table S3). There were no associations between time-domain parameters and MMSE scores (Table 3).

In terms of the MoCA score, univariable analysis showed that the HF norm was associated with a better MoCA score (β = 0.069; 95% CI: 0.014, 0.124; *p* = 0.014), whereas the LF/HF ratio was associated with a worse MoCA score (β = −0.738; 95% CI: −1.302, −0.174; *p* = 0.011). After adjusting for age, sex, BMI, education, hypertension, diabetes mellitus, dyslipidemia, and history of stroke, multiple linear regression analyses showed that a higher HF norm was independently associated with a better MoCA score (β = 0.061; 95% CI: 0.017, 0.105; *p* = 0.007). Conversely, a higher LF/HF ratio was independently associated with worse MoCA scores (β = −0.691; 95% CI: −1.134, −0.248; *p* = 0.003) (Table 3). Subgroup analyses showed that the significant associations between frequency domain parameters and MoCA score were only observed in patients with age <75 (HF norm: β = 0.063; 95% CI: 0.012, 0.114; *p* = 0.016; LF/HF ratio: β = −0.619; 95% CI: −1.117, −0.121; *p* = 0.015), patients with hypertension (HF norm: β = 0.055; 95% CI: 0.005, 0.104; *p* = 0.031; LF/HF ratio: β = −0.628; 95% CI: −1.108, −0.147; *p* = 0.001), patients without diabetes (HF norm: β = 0.075; 95% CI: 0.025, 0.125; *p* = 0.004; LF/HF ratio: β = −0.692; 95% CI: −1.204, −0.180; *p* = 0.009), and patients without history of stroke (HF norm: β = 0.074; 95% CI: 0.021, 0.128; *p* = 0.008; LF/HF ratio: β = −0.689; 95% CI: −1.200, −0.179; *p* = 0.009) (Supplementary Table S3). No significant association was found between time-domain parameters and MoCA scores (Table 3).

## 4. Discussion

In this study, our findings demonstrate that higher levels of HF norm were independently associated with better performance on the MMSE and MoCA tests, while higher levels of LF/HF ratio were independently associated with worse performance on the MMSE and MoCA tests.

In our study, univariable analysis showed significant differences in HF norm and LF/HF ratio between patients with and without cognitive dysfunction, indicating that patients with cognitive dysfunction had lower parasympathetic tone than those without cognitive dysfunction. Spearman correlation analyses showed that the HF norm was positively correlated with cognitive performance, whereas the LF/HF ratio was negatively correlated with cognitive performance. After adjusting for age, sex, education, and health conditions, the significant associations between frequency domain parameters and cognitive performance still remained, further suggesting better parasympathetic function might be associated with better cognitive performance, while sympatho-vagal imbalance (sympathetic dominance) might be associated with worse cognitive performance.

Previous studies have reported inconsistent results regarding the association between parasympathetic function and cognitive function [17,18,28,29,30]. In our study, the normalized HF, which represents the parasympathetic percentage, was positively and significantly associated with global cognitive function after adjusting for confounding covariates in our study. In line with our findings, a cross-sectional study conducted among 311 adults from the Women’s Health and Aging Study I showed that reduced HF was significantly associated with 6.7 times greater odds of cognitive impairment (odds ratio = 6.74; 95% CI: 2.27, 20.00; *p* = 0.001) in older disabled women in the community [17]. Nevertheless, some studies have suggested no relationship between vagal activity and cognitive performance. Data consisted of 1685 atrial fibrillation patients from the Swiss Atrial Fibrillation Cohort showed no direct association between HF and MoCA score in patients with atrial fibrillation (β = −0.007; 95% CI: −0.648, 0.634; *p* = 0.982) and in patients with sinus rhythm (β = 0.047; 95% CI: −0.192, 0.287; *p* = 0.699) [29]. Similarly, a study on an elderly patient representative of outpatients in a real-life setting reported that HF was not significantly associated with performance on MMSE (β = −0.049, *p* = 0.602) and MoCA (β = 0.026, *p* = 0.775) tests [18]. Moreover, cross-sectional analysis of wave 1 data from the Irish longitudinal study on ageing suggested that lower quintiles of HF was not significantly associated with lower MoCA scores (*p* = 0.7) [28]. In a prospective cohort study of 5375 participants from the Whitehall II Study, the HF was not independently associated with memory (OR = 0.98, 95% CI: 0.88–1.10) and decline in memory (OR = 1.01, 95% CI: 0.91–1.11) in a middle-aged population [30]. These interstudy differences, to a certain extent, may be due to different study populations, different control groups, and different/unstandardized assessments and analytic protocols of HRV data. In this study, we found gentle association between RMSSD and global cognitive function. Although it was discrepant with some previous studies [16,31], it provided evidences to support the conjecture that the balance of the ANS system might be more important than the absolute function of the ANS sub-system to correlate cognitive function. ANS balance reflects the whole homeostasis of the body; however, the absolute value of HRV has not been well defined all along the time because HRV is influenced by many physiological and psychological factors and related with individualized characters. Furthermore, 24 h time-domain analysis of HRV may provide more information including circadian rhythm of HRV to correlate cognitive function. Future studies with standardized designs are urgently needed to explore the association between parasympathetic modulation and cognitive performance.

Our findings are consistent with those of previous studies on the association between a higher LF/HF ratio, an index of sympatho-vagal imbalance toward vagal withdraw or sympathetic activation, and cognitive dysfunction [18,28,32,33,34]. For example, cross-sectional results from patients with Alzheimer’s disease showed that the LF/HF ratio was negatively associated with the performance on the MMSE (β = −0.31; 95% CI: −1.52, −0.27; *p* = 0.006) and cognitive composite scores (β = −0.26; 95% CI: −2.13, −0.17; *p* = 0.020) [32]. Results from patients with diabetes showed that an increased LF/HF ratio was significantly associated with worse cognitive performance, as assessed by P300 (β = 0.356; 95% CI: 0.012, 2.072; *p* = 0.007) [33]. Nevertheless, our results differed from those of a recent study conducted among elderly outpatients in a real-life setting, which found a positive correlation between a higher LF/HF ratio and better cognitive performance evaluated by MMSE and MoCA tests, possibly because of the older age of participants (mean age = 73.5 years old) and healthier conditions of control group [18]. In the Irish longitudinal study on aging wave one, which was an older cohort of non-demented community dwelling, there was a dose–response association between a high LF/HF ratio and a high MoCA score during spontaneous and paced breathing periods in community dwelling non-demented people [28]. Possible explanations for those discrepancies were differences in study subjects and control group. In addition, a study conducted among healthy females showed no association between the LF/HF ratio and performance on the Wechsler Memory Scale-Revised, a test of memory [34]; however, this study assessed different domains of memory function than the current, including verbal memory, visual memory, general memory, attention/concentration, and delayed recall. Moreover, the concerned study is composed of healthy female subjects only, unlike our study, which consists of male and female patients with a higher proportion of multiple risk factors (e.g., hypertension, dyslipidemia and diabetes mellitus). Differences in study samples, control groups, and in cognitive domains explored may contribute to the interstudy discrepancy in the association between the LF/HF ratio and cognitive function.

Several mechanisms may contribute to the association between HRV and cognitive performance. The autonomic nervous system is crucial in regulating blood flow and sustaining adequate and constant cerebral perfusion [35]. Altered HRV, as a reflection of autonomic dysfunction, has been associated with poor baroreflex sensitivity [36,37], which may result in increased blood pressure variability and suboptimal cerebral perfusion. Higher blood pressure variability is associated with cognitive decline and with structural brain lesions related to hypertension, such as WMH, and to stroke, such as lacunar infarctions [38,39]. Furthermore, cardiovascular risk factors such as hypertension and diabetes mellitus have been linked to both cognitive decline and depressed HRV [40,41], indicating a possible role in the association between HRV and cognitive impairment. However, the association remained even after adjusting for several cardiovascular risk factors in our study, suggesting that these variables do not account for the observed significant association between HRV and cognitive performance. HRV is also linked to cardiovascular morbidity, which in turn might result in impaired cognition [42]. Moreover, neurodegenerative changes may influence autonomic function through altered autonomic pathways. Abnormal HRV may result from autonomic dysfunction secondary to central nervous system changes during the preclinical phase of dementia [43]. Previous studies have shown that a network comprising the insular cortex, amygdala, hypothalamus, and nucleus tractus solitarius is essential in regulating autonomic function [44] and is also associated with cognitive function [45].

To the best of our knowledge, this is the first report to investigate the association between HRV and cognitive performance among patients with CSVD, a group at a high risk of cognitive decline. We used two reliable and validated screening tools for cognitive impairment that provide a rigorous assessment of global cognitive function. Moreover, we used both time-domain and frequency-domain HRV parameters to reflect the absolute function and balance of autonomic systems. However, the present study has several limitations. First, it is challenging to establish the temporality of the relationship because of the cross-sectional and observational nature of the study. Future study will focus on predictive designs that help examine the causal inference of autonomic function on global cognitive function. Second, this study included a relatively small sample size of participants; therefore, the findings of our study need to be confirmed in larger longitudinal studies in the future. Third, the participants in our study were CSVD population at a high risk of cognitive dysfunction, so we will include healthy age-matched controls in future studies in order to make a solid interpretation. Finally, we used short-term HRV analyses, which is time-consuming and convenient; however, the circadian features of ANS and HRV were not obtained. 24-h or longer time of monitoring are expected in future studies.

## 5. Conclusions

The HF norm was positively associated with cognitive performance, whereas the LF/HF ratio was negatively associated with cognitive performance among patients with CSVD. Larger-scale prospective studies are warranted to confirm these findings and further clarify the nature of these associations.

## Figures and Tables

**Table 1:** Baseline characteristics of patients with CSVD.

	All CSVD (*n* = 117)
Age, mean (SD)	59.53 (11.79)
Women, *n* (%)	46 (39.32)
BMI, mean (SD)	25.32 (3.29)
Education, *n* (%)	
College degree or above	30 (25.64)
Middle school	69 (58.97)
Elementary school or below	18 (15.38)
Risk factors, *n* (%)	
Hypertension	95 (81.20)
Diabetes mellitus	48 (41.03)
Dyslipidemia	105 (89.74)
Current smoking	37 (31.62)
Drinking	29 (24.79)
Medical history, *n* (%)	
Stroke	52 (44.44)
Coronary heart disease	17 (14.53)
MMSE, median (Q1, Q3)	26.00 (22.00, 27.00)
MoCA, median (Q1, Q3)	21.00 (17.00, 25.00)

BMI, body mass index; CSVD, cerebral small vessel disease; MMSE, Mini-Mental-State Examination; MoCA, Montreal Cognitive Assessment; SD, standard deviation.

**Table 2:** HRV parameters of patients with CSVD [Median (Q1, Q3)].

	All CSVD (*n* = 117)	Normal Cognitive Function Defined by MMSE (*n* = 76)	Impaired Cognitive Function Defined by MMSE (*n* = 41)	*p* Value	Normal Cognitive Function Defined by MoCA (*n* = 18)	Impaired Cognitive Function Defined by MoCA (*n* = 98)	*p* Value
Time domain							
SDNN (ms)	17.98 (12.31, 23.52)	17.79 (12.71, 23.11)	17.98 (11.27, 26.35)	0.882	20.10 (15.44, 25.59)	17.27 (12.05, 23.52)	0.250
RMSSD (ms)	19.37 (12.25, 28.20)	19.23 (12.86, 27.94)	19.85 (10.73, 28.77)	0.656	22.97 (17.03, 28.20)	18.96 (11.25, 28.37)	0.234
Frequency domain							
HF norm (n.u.)	50.13 (39.46, 64.99)	57.23 (42.68, 67.46)	42.95 (29.05, 52.59)	<0.001	56.31 (45.39, 68.75)	48.22 (39.35, 64.08)	0.225
LF/HF ratio	0.99 (0.54, 1.53)	0.75 (0.48, 1.34)	1.33 (0.90, 2.44)	<0.001	0.78 (0.45, 1.20)	1.07 (0.56, 1.54)	0.225

CSVD, cerebral small vessel disease; HF, high frequency; HRV, heart rate variability; LF, low frequency; RMSSD, root mean squares of successive differences of normal/normal intervals; SDNN, standard deviation of the normal/normal intervals.

**Table 3:** Associations between HRV parameters and MMSE and MoCA scores among total CSVD patients.

	MMSE Scores				MoCA Scores			
	Unadjusted		Adjusted		Unadjusted		Adjusted	
	β (95% CI)	*p* Value	β (95% CI)	*p* Value	β (95% CI)	*p* Value	β (95% CI)	*p* Value
Time domain								
SDNN (ms)	−0.520 (−1.925, 0.884)	0.465	−0.610 (−1.832, 0.613)	0.325	−0.167 (−1.833, 1.498)	0.843	−0.534 (−1.918, 0.850)	0.446
RMSSD (ms)	−0.106 (−1.301, 1.090)	0.861	−0.061 (−1.109, 0.986)	0.908	0.110 (−1.304, 1.524)	0.878	0.049 (−1.132, 1.231)	0.934
Frequency domain								
HF norm (n.u.)	0.057 (0.010, 0.104)	0.018	0.051 (0.012, 0.090)	0.011	0.069 (0.014, 0.124)	0.014	0.061 (0.017, 0.105)	0.007
LF/HF ratio	−0.576 (−1.058, −0.093)	0.020	−0.492 (−0.893, −0.092)	0.017	−0.738 (−1.302, −0.174)	0.011	−0.691 (−1.134, −0.248)	0.003

Absolute values of HRV were transformed by natural logarithm before entering model. Values were adjusted for age, gender, BMI, education, hypertension, diabetes mellitus, dyslipidemia, and history of stroke. CI: confidence interval; CSVD, cerebral small vessel disease; HF, high frequency; HRV, heart rate variability; LF, low frequency; MMSE, Mini-Mental-State Examination; MoCA, Montreal Cognitive Assessment; RMSSD, root mean squares of successive differences of normal/normal intervals; SDNN, standard deviation of the normal/normal intervals.

## Data Availability

The data are available to researchers on reasonable request from the corresponding author for the purpose of reproducing results or replicating the procedure.

## Supplementary Materials

The following supporting information can be downloaded at: https://www.mdpi.com/article/10.3390/brainsci13020344/s1, Table S1: Spearman correlation between HRV parameters and MMSE and MoCA scores among patients with CSVD; Table S2: Spearman correlation between HRV parameters and MMSE and MoCA scores among different subgroup patients with CSVD; Table S3: Subgroup analyses of associations between HRV parameters and MMSE and MoCA scores; Figure S1: QQ plots of continuous variables.

## Abbreviations

BMI, body mass index; CI: confidence interval; CSVD, cerebral small vessel diseases; ECG, electrocardiogram; HF, high frequency; HRV, heart rate variability; IQR, interquartile range; LF, low frequency; MMSE, Mini-Mental State Examination; MoCA, Montreal Cognitive Assessment; MRI, magnetic resonance imaging; RMSSD, root mean squares of successive differences of normal-normal intervals; SDNN, standard deviation of the normal-normal intervals; STRIVE, Standards for Reporting Vascular Changes on Neuroimaging; WMH, white matter hyperintensity.
