# Supplementary material for: Associations between Autonomic Function and Cognitive Performance among Patients with Cerebral Small Vessel Disease
Source: Brain Sci. 2023 Feb 17;13(2):344. doi: 10.3390/brainsci13020344 (PMC9954276; doi:10.3390/brainsci13020344)
Supplement: Supplementary file 1 [file brainsci-13-00344-s001.zip › brainsci-2186170-supplementary/Supplementary File_Proofed.docx]

**Supplementary File**

Associations between Autonomic Function and Cognitive Performance among Patients with Cerebral Small Vessel Disease

Guoliang Hu ^1,2,3,4^, Jean-Paul Collet ^5^, Mengxi Zhao ^1,2,3,4^, Yao Lu ^1,2,3,4^ and Yilong Wang ^1,2,3,4,6,^*

^1^ Department of Neurology, Beijing Tiantan Hospital, Capital Medical University, Beijing 100070, China; guoliang_hu@163.com (G.H.); mxrdf0909@163.com (M.Z.); luyao970131@163.com (Y.L.)

^2^ China National Clinical Research Center for Neurological Diseases, Beijing 100070, China

^3^ Advanced Innovation Center for Human Brain Protection, Capital Medical University, Beijing 100070, China

^4^ National Center for Neurological Diseases, Beijing 100070, China

^5^ Department of Medicine, The University of British Columbia, Vancouver, BC V6T 1Z4, Canada; jcollet@bcchr.ca (J.C.)

^6^ Chinese Institute for Brain Research, Beijing 102206, China

***** Correspondence: yilongwang528@163.com; Tel.: 0086-010-59975029

**Methods**

Hypertension was deﬁned as having a previous or new diagnosis of hypertension, receiving antihypertensive therapy, or having a systolic blood pressure≥140 mmHg or diastolic blood pressure≥90 mmHg at admission. Diabetes was defined as having a history of diabetes, receiving anti-diabetic treatment, or having a hemoglobin A1c level of ≥6.5%, or having diabetes listed as a discharge diagnosis in the medical records. Dyslipidemia was deﬁned as having a previous or new diagnosis of dyslipidemia, or receiving lipid-lowering therapy. Other clinical history of diseases, including history of stroke and coronary heart disease were defined based on the original medical records. Body mass index was defined as weight/height^2^ in kg/m^2^. Current smoking was defined as smoking≥1 cigarette per day on average within one year preceding the current hospitalization episode.

The structural magnetic resonance imaging included the following: whole brain T1-weighted image [repetition time (TR), 2300 ms; inversion time (TI), 900 ms; echo time (TE), 2.3 ms; field of view (FOV), 256×256×196 mm^3^; voxel size, 1.0×1.0×1.0 mm^3^; flip angle (FA), 8°] for anatomic reference, T2-weighted fluid-attenuated inversion recovery image (TR, 5000 ms; TI, 1800 ms; TE, 386 ms; FOV, 256×256×196 mm^3^; voxel size, 1.0×1.0×1.0 mm^3^; FA, 40°) for assessment of white matter hyperintensities, lacunes and enlarged perivascular space, diffusion-weighted imaging sequence (TR, 6100 ms; TE, 65 ms; FOV, 184×226×143 mm^3^; voxel size, 1.6×2.0×5.0 mm^3^; diffusion sensitizing gradient directions b, 1200 sec/mm^2^) for assessment of new lacunar infarcts, and susceptibility-weighted imaging sequence (TR, 29; TE, 20 ms; FOV, 210×240×159 mm^3^; voxel size, 0.5×0.5×1.5 mm^3^; FA, 15°) for assessment of microbleeds.

Using our population of a total of 117 patients for a linear regression analysis at a significance level of 0.05, we achieved a 99% power to detect the association between HRV and MMSE score and MoCA score (PASS 11.0, Power Analysis and Sample Size Software, NCSS, LLC, USA).

**Supplementary Table S1.** Spearman correlation between HRV parameters and MMSE and MoCA scores among patients with CSVD

|  | MMSE scores | | MoCA scores | |
| --- | --- | --- | --- | --- |
|  | rs | P Value | rs | P Value |
| Time domain |  |  |  |  |
| SDNN (ms) | 0.006 | 0.947 | 0.063 | 0.501 |
| RMSSD (ms) | 0.026 | 0.779 | 0.069 | 0.465 |
| Frequency domain |  |  |  |  |
| HF norm (n.u.) | 0.217 | 0.019 | 0.252 | 0.006 |
| LF/HF ratio | -0.217 | 0.019 | -0.252 | 0.006 |

CSVD, cerebral small vessel disease; HF, high frequency; HRV, heart rate variability; LF, low frequency; MMSE, Mini-Mental State Examination; MoCA, Montreal Cognitive Assessment; RMSSD, root mean squares of successive differences of normal/normal intervals; SDNN, standard deviation of the normal/normal intervals.

**Supplementary Table S2.** Spearman correlation between HRV parameters and MMSE and MoCA scores among different subgroup patients with CSVD

|  | MMSE Scores | | MoCA Scores | |
| --- | --- | --- | --- | --- |
|  | rs | P Value | rs | P Value |
| Age <75 |  |  |  |  |
| SDNN (ms) | -0.016 | 0.873 | 0.030 | 0.760 |
| RMSSD (ms) | 0.021 | 0.829 | 0.054 | 0.588 |
| HF norm (n.u.) | 0.235 | 0.016 | 0.267 | 0.006 |
| LF/HF ratio | -0.235 | 0.016 | -0.267 | 0.006 |
| Age ≥75 |  |  |  |  |
| SDNN (ms) | -0.042 | 0.897 | 0.049 | 0.879 |
| RMSSD (ms) | -0.102 | 0.753 | 0.021 | 0.948 |
| HF norm (n.u.) | -0.116 | 0.720 | -0.035 | 0.913 |
| LF/HF ratio | 0.116 | 0.720 | 0.035 | 0.913 |
| Without hypertension |  |  |  |  |
| SDNN (ms) | 0.372 | 0.088 | 0.368 | 0.092 |
| RMSSD (ms) | 0.330 | 0.133 | 0.161 | 0.473 |
| HF norm (n.u.) | 0.193 | 0.390 | 0.131 | 0.562 |
| LF/HF ratio | -0.193 | 0.390 | -0.131 | 0.562 |
| With hypertension |  |  |  |  |
| SDNN (ms) | -0.054 | 0.602 | 0.016 | 0.877 |
| RMSSD (ms) | -0.035 | 0.734 | 0.030 | 0.773 |
| HF norm (n.u.) | 0.217 | 0.035 | 0.262 | 0.011 |
| LF/HF ratio | -0.217 | 0.035 | -0.262 | 0.011 |
| Without diabetes |  |  |  |  |
| SDNN (ms) | 0.130 | 0.287 | 0.298 | 0.013 |
| RMSSD (ms) | 0.182 | 0.134 | 0.290 | 0.015 |
| HF norm (n.u.) | 0.273 | 0.023 | 0.296 | 0.014 |
| LF/HF ratio | -0.273 | 0.023 | -0.296 | 0.014 |
| With diabetes |  |  |  |  |
| SDNN (ms) | -0.167 | 0.255 | -0.280 | 0.057 |
| RMSSD (ms) | -0.193 | 0.188 | -0.266 | 0.071 |
| HF norm (n.u.) | 0.108 | 0.466 | 0.145 | 0.331 |
| LF/HF ratio | -0.108 | 0.466 | -0.145 | 0.331 |
| Without history of stroke |  |  |  |  |
| SDNN (ms) | 0.045 | 0.723 | 0.060 | 0.635 |
| RMSSD (ms) | 0.015 | 0.905 | 0.024 | 0.852 |
| HF norm (n.u.) | 0.231 | 0.064 | 0.255 | 0.040 |
| LF/HF ratio | -0.231 | 0.064 | -0.255 | 0.040 |
| With history of stroke |  |  |  |  |
| SDNN (ms) | -0.111 | 0.432 | -0.121 | 0.396 |
| RMSSD (ms) | -0.058 | 0.683 | -0.063 | 0.658 |
| HF norm (n.u.) | 0.168 | 0.233 | 0.198 | 0.164 |
| LF/HF ratio | -0.168 | 0.233 | -0.198 | 0.164 |

CSVD, cerebral small vessel disease; HF, high frequency; HRV, heart rate variability; LF, low frequency; MMSE, Mini-Mental State Examination; MoCA, Montreal Cognitive Assessment; RMSSD, root mean squares of successive differences of normal/normal intervals; SDNN, standard deviation of the normal/normal intervals.

**Supplementary Table S3.** Subgroup analyses of associations between HRV parameters and MMSE and MoCA scores

|  | MMSE Scores | | | MoCA Scores | | |
| --- | --- | --- | --- | --- | --- | --- |
|  | β | 95% CI | P Value | β | 95% CI | P Value |
| Age<75 |  |  |  |  |  |  |
| SDNN (ms) | -0.633 | -1.988, 0.723 | 0.357 | -0.758 | -2.332, 0.817 | 0.342 |
| RMSSD (ms) | -0.201 | -1.376, 0.974 | 0.735 | -0.389 | -1.749, 0.972 | 0.572 |
| HF norm (n.u.) | 0.059 | 0.015, 0.103 | 0.009 | 0.063 | 0.012, 0.114 | 0.016 |
| LF/HF ratio | -0.466 | -0.901, -0.030 | 0.036 | -0.619 | -1.117, -0.121 | 0.015 |
| Age≥75 |  |  |  |  |  |  |
| SDNN (ms) | 3.016 | -10.494, 16.526 | 0.569 | 2.101 | -11.736, 15.938 | 0.695 |
| RMSSD (ms) | 0.138 | -7.233, 7.510 | 0.961 | -0.026 | -7.398, 7.346 | 0.993 |
| HF norm (n.u.) | -0.068 | -0.231, 0.094 | 0.307 | -0.084 | -0.232, 0.063 | 0.189 |
| LF/HF ratio | 0.934 | -2.384, 4.252 | 0.478 | 1.297 | -1.777, 4.370 | 0.307 |
| Without hypertension |  |  |  |  |  |  |
| SDNN (ms) | 0.593 | -6.851, 8.037 | 0.866 | -1.076 | -10.042, 7.890 | 0.800 |
| RMSSD (ms) | 2.899 | -3.482, 9.280 | 0.344 | 1.553 | -6.369, 9.475 | 0.679 |
| HF norm (n.u.) | 0.083 | -0.019, 0.185 | 0.102 | 0.090 | -0.036, 0.216 | 0.148 |
| LF/HF ratio | -1.195 | -2.197, -0.193 | 0.023 | -1.245 | -2.529, 0.040 | 0.057 |
| With hypertension |  |  |  |  |  |  |
| SDNN (ms) | -0.963 | -2.228, 0.303 | 0.134 | -0.881 | -2.311, 0.548 | 0.224 |
| RMSSD (ms) | -0.468 | -1.560, 0.624 | 0.396 | -0.286 | -1.515, 0.944 | 0.646 |
| HF norm (n.u.) | 0.035 | -0.010, 0.080 | 0.123 | 0.055 | 0.005, 0.104 | 0.031 |
| LF/HF ratio | -0.336 | -0.778, 0.107 | 0.136 | -0.628 | -1.108, -0.147 | 0.011 |
| Without diabetes |  |  |  |  |  |  |
| SDNN (ms) | 1.594 | -0.308, 3.496 | 0.099 | 2.916 | 0.723, 5.109 | 0.010 |
| RMSSD (ms) | 1.630 | 0.241, 3.020 | 0.022 | 2.307 | 0.683, 3.932 | 0.006 |
| HF norm (n.u.) | 0.066 | 0.025, 0.108 | 0.002 | 0.075 | 0.025, 0.125 | 0.004 |
| LF/HF ratio | -0.516 | -0.951, -0.081 | 0.021 | -0.692 | -1.204, -0.180 | 0.009 |
| With diabetes |  |  |  |  |  |  |
| SDNN (ms) | -2.034 | -3.624, -0.444 | 0.014 | -2.731 | -4.434, -1.028 | 0.002 |
| RMSSD (ms) | -1.699 | -3.226, -0.173 | 0.030 | -2.101 | -3.779, -0.422 | 0.016 |
| HF norm (n.u.) | 0.018 | -0.065, 0.101 | 0.657 | 0.045 | -0.046, 0.135 | 0.324 |
| LF/HF ratio | -0.328 | -1.249, 0.594 | 0.476 | -0.832 | -1.814, 0.150 | 0.094 |
| Without history of stroke |  |  |  |  |  |  |
| SDNN (ms) | -0.761 | -2.118, 0.596 | 0.266 | -0.595 | -2.419, 1.229 | 0.516 |
| RMSSD (ms) | -0.088 | -1.260, 1.084 | 0.881 | 0.261 | -1.302, 1.823 | 0.739 |
| HF norm (n.u.) | 0.057 | 0.017, 0.097 | 0.006 | 0.074 | 0.021, 0.128 | 0.008 |
| LF/HF ratio | -0.420 | -0.811, -0.029 | 0.036 | -0.689 | -1.200, -0.179 | 0.009 |
| With history of stroke |  |  |  |  |  |  |
| SDNN (ms) | -0.865 | -3.364, 1.633 | 0.489 | -1.162 | -3.691, 1.366 | 0.359 |
| RMSSD (ms) | -0.532 | -2.696, 1.631 | 0.622 | -0.925 | -3.114, 1.264 | 0.399 |
| HF norm (n.u.) | 0.034 | -0.051, 0.120 | 0.422 | 0.032 | -0.051, 0.114 | 0.441 |
| LF/HF ratio | -0.610 | -1.676, 0.456 | 0.255 | -0.625 | -1.642, 0.391 | 0.221 |

HF, high frequency; HRV, heart rate variability; LF, low frequency; MMSE, Mini-Mental State Examination; MoCA, Montreal Cognitive Assessment; RMSSD, root mean squares of successive differences of normal/normal intervals; SDNN, standard deviation of the normal/normal intervals.


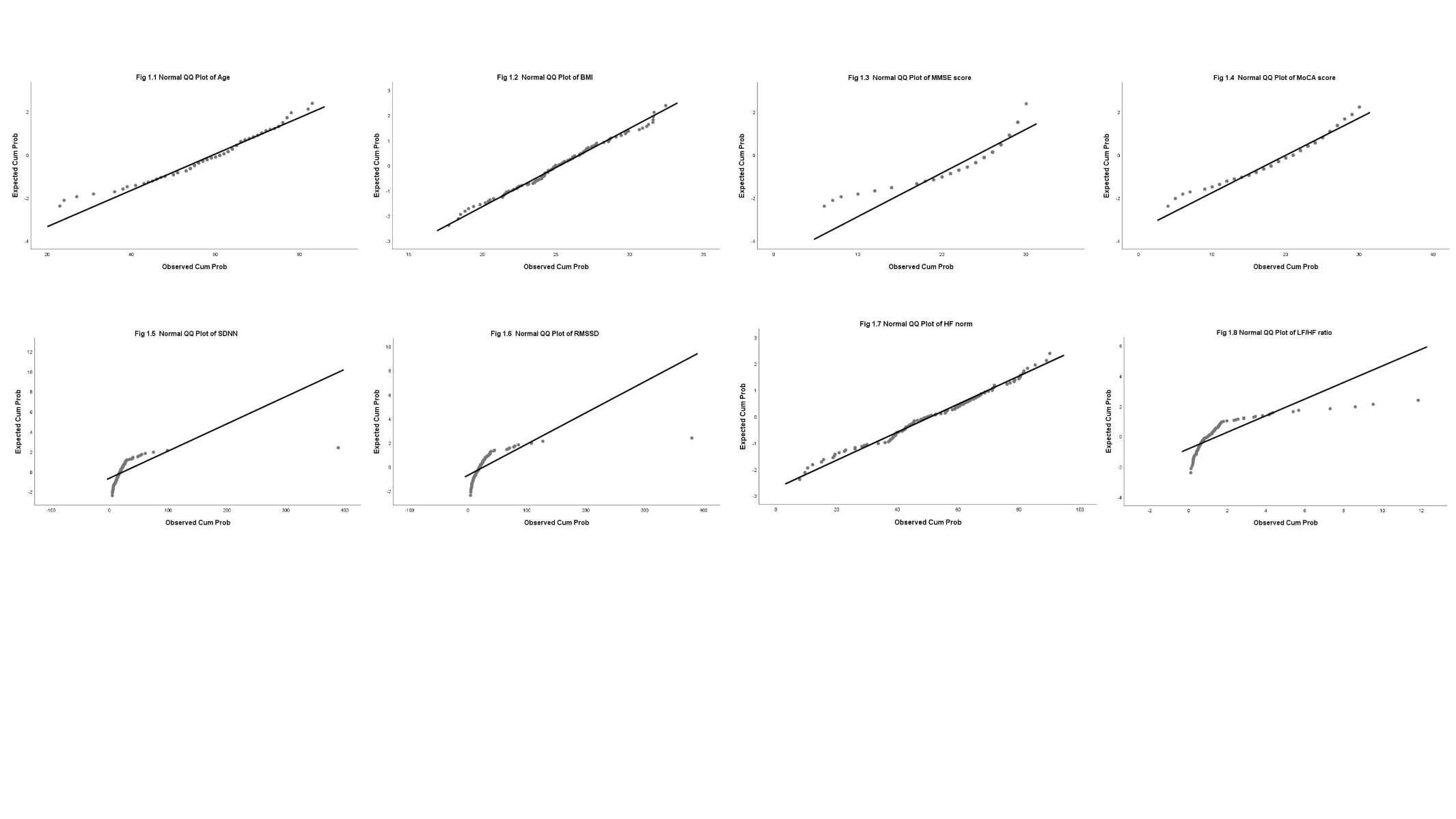


**Supplementary Figure S1 QQ plots of continuous variables**

BMI, body mass index; HF, high frequency; LF, low frequency; MMSE, Mini-Mental State Examination; MoCA, Montreal Cognitive Assessment; RMSSD, the root mean squares of successive differences of normal/normal intervals; SDNN, standard deviation of the normal/normal intervals.
